# Placental DNA hypomethylation in association with particulate air pollution in early life

**DOI:** 10.1186/1743-8977-10-22

**Published:** 2013-06-07

**Authors:** Bram G Janssen, Lode Godderis, Nicky Pieters, Katrien Poels, Michał Kiciński, Ann Cuypers, Frans Fierens, Joris Penders, Michelle Plusquin, Wilfried Gyselaers, Tim S Nawrot

**Affiliations:** 1 Centre for Environmental Sciences, Hasselt University, Agoralaan gebouw D, Diepenbeek, 3590, Belgium; 2 Department of Public Health, Occupational, Environmental & Insurance Medicine, Leuven University (KU Leuven), Leuven, Belgium; 3 Idewe, External Service for Prevention and Protection at Work, Heverlee, Belgium; 4 Belgian Interregional Environment Agency, Brussels, Belgium; 5 Laboratory of Clinical Biology, East-Limburg Hospital, Genk, Belgium; 6 Biomedical Research Institute, Hasselt University, Diepenbeek, Belgium; 7 Department of Obstetrics, East-Limburg Hospital, Genk, Belgium

**Keywords:** Fetal development, DNA methylation, Particulate matter, Placental tissue

## Abstract

**Background:**

There is evidence that altered DNA methylation is an important epigenetic mechanism in prenatal programming and that developmental periods are sensitive to environmental stressors. We hypothesized that exposure to fine particles (PM_2.5_) during pregnancy could influence DNA methylation patterns of the placenta.

**Methods:**

In the ENVIR*ON*AGE birth cohort, levels of 5’-methyl-deoxycytidine (5-mdC) and deoxycytidine (dC) were quantified in placental DNA from 240 newborns. Multiple regression models were used to study placental global DNA methylation and *in utero* exposure to PM_2.5_ over various time windows during pregnancy.

**Results:**

PM_2.5_ exposure during pregnancy averaged (25^th^-75^th^ percentile) 17.4 (15.4-19.3) μg/m^3^. Placental global DNA methylation was inversely associated with PM_2.5_ exposures during whole pregnancy and relatively decreased by 2.19% (95% confidence interval [CI]: -3.65, -0.73%, *p =* 0.004) for each 5 μg/m^3^ increase in exposure to PM_2.5_. In a multi-lag model in which all three trimester exposures were fitted as independent variables in the same regression model, only exposure to PM_2.5_ during trimester 1 was significantly associated with lower global DNA methylation (-2.13% per 5 μg/m^3^ increase, 95% CI: -3.71, -0.54%, *p* = 0.009). When we analyzed shorter time windows of exposure within trimester 1, we observed a lower placental DNA methylation at birth during all implantation stages but exposure during the implantation range (6-21d) was strongest associated (-1.08% per 5 μg/m^3^ increase, 95% CI: -1.80, -0.36%, *p* = 0.004).

**Conclusions:**

We observed a lower degree of placental global DNA methylation in association with exposure to particulate air pollution in early pregnancy, including the critical stages of implantation. Future studies should elucidate genome-wide and gene-specific methylation patterns in placental tissue that could link particulate exposure during *in utero* life and early epigenetic modulations.

## Background

The human placenta forms the interface between fetal and maternal circulation and plays a critical role in the regulation of fetal growth and development through controlled nutrient supply. Fetal adaptations and developmental plasticity arising from perturbations in utero-placental exchange to meet fetal requirements “program” the fetus for an increased risk of developing cardiovascular disease and diabetes in adult life [1,2]. Epigenetic modifications, described as heritable changes in genes function that cannot be not be explained by changes in the underlying DNA sequence, are believed to play an essential role in this process. With exception of imprinted genes, all DNA methylation patterns are established during embryogenesis, and play an important role in gene regulation which could comprise a biologically plausible link between *in utero* exposures and disease risks during adulthood [3].

Several studies support evidence of detrimental effects of particulate matter (PM) on the health outcomes of fetuses [4,5], neonates [6,7] and is later in life associated with cardiovascular morbidity and mortality [8-10]. DNA methylation is, besides histone modification and non-coding RNAs, a well-characterized epigenetic modification that may provide an attractive mechanism linking particulate air pollution in early life and health consequences in adulthood [11]. Indeed, data from the Dutch Hunger Winter (1944–45) indicate that prenatal environmental conditions can cause epigenetic changes in humans that persist throughout life [12]. In addition to these observational data, animal studies showed that certain transient environmental influences during *in utero* life could produce persistent changes in epigenetic marks that have life-long consequences [13,14].

Alterations in DNA methylation patterns are mediated by several factors and have been associated with many different health outcomes [15]. Evidence from animal and human studies in adults indicate that particulate air pollution may affect global and gene-specific methylation [16-19]. A number of studies [20-22] describe DNA methylation patterns in placental tissue but the association between particulate air pollution and DNA methylation in placental tissue has never been investigated.

Within the ENVIR*ON*AGE birth cohort, we determined whether exposure to ambient particulate matter (PM_2.5_) during different periods of prenatal life is associated with differences in global DNA methylation of placental tissue at birth.

## Results

### Characteristics and exposure levels of the study population

The study included 240 mother-newborn pairs (mean maternal age, 29.1 yr; range, 18–42 yr). Demographic and prenatal lifestyle factors are reported in Table 1. Briefly, mean (± SD) pre-gestational BMI of the participating mothers was 24.4 (± 4.5) kg/m^2^. 39 mothers (16.2%) reported to have smoked during pregnancy and smoked a mean number of 7.2 (± 4.2) cigarettes per day. Most women (67.6%, *n* = 162) never smoked cigarettes. More than 50% of the mothers were high educated. The newborn population, including 130 girls (54.2%), had a mean gestational age of 39.2 weeks (range, 35–42), 96.2% were term born infants and included a vast majority of primiparous (53.3%, *n* = 128) or secundiparous (36.7%, *n* = 88) newborns.

**Table 1:** **Characteristics of mother-newborn pairs (*n*** **= 240)**

**Characteristics**	**Mean ± SD or range and number (%)**
Maternal	
Age, y	29.1 (18–42)
Pre-gestational BMI, kg/m^2^	24.4 ± 4.5
Net weight gain, kg	14.6 ± 6.4
Maternal education	
Low	30 (12.5%)
Middle	84 (35.0%)
High	126 (52.5%)
Smoking	
Never-smoker	162 (67.6%)
Past-smoker	39 (16.2%)
Smoker	39 (16.2%)
Acetaminophen	
No	136 (56.7%)
Yes	104 (43.3%)
Alcohol^a^	
No	194 (82.5%)
Yes	41 (17.5%)
Parity	
1	128 (53.3%)
2	88 (36.7%)
≥ 3	24 (10.0%)
Apparent temperature, °C	
Trimester 1	9.4 ± 6.0
Trimester 2	8.3 ± 6.2
Trimester 3	8.6 ± 5.9
Newborn	
Newborn’s gender	
Male	110 (45.8%)
Female	130 (54.2%)
Ethnicity^b^	
European	206 (86.5%)
Non-European	32 (13.5%)
Gestational age, w	39.2 (35–42)
Season^c^	
Fall	56 (23.3%)
Winter	60 (25.0%)
Spring	51 (21.3%)
Summer	73 (30.4%)
Apgar score after 5 min	
7	2 (0.8%)
8	11 (4.6%)
9	74 (30.8%)
10	153 (63.8%)
Birth weight, g	3400.8 ± 422.3
Birth length, cm	50.2 ± 1.9
Placental global DNA methylation, %	4.6 ± 0.4

^a^Data available for 235 and ^b^238 subjects.

^c^Season determined for date of conception.

Table 2 presents the mean outdoor exposure to PM_2.5_ averaged for the implantation windows and for each of the three trimesters of pregnancy. Average (25^th^-75^th^ percentile) trimester-specific PM_2.5_ exposure was 16.7 (12.3-20.0) μg/m^3^ for the first trimester, 17.4 (12.0-22.1) μg/m^3^ for the second trimester, 18.2 (12.8-22.9) μg/m^3^ for the third trimester and 17.4 (15.4-19.3) μg/m^3^ for the whole pregnancy. Nitrogen dioxide (NO_2_) and maximum 8-hour average ozone (O_3_) for the specific exposure windows are presented in Additional file 1: Table S1.

**Table 2:** **Exposure characteristics (*n*** **= 240)**

**PM**_**2.5**_**, μg/m**^**3**^	**Mean**	**SD**	**25**^**th **^**percentile**	**75**^**th **^**percentile**
Pre-implantation (1-5d)	16.9	11.0	10.4	19.3
Implantation (6-12d)	16.9	9.8	10.1	20.1
Implantation range (6-21d)	16.7	8.1	11.3	20.4
Post-implantation (22-28d)	17.3	10.4	10.1	20.4
Trimester 1 (1-13w)	16.7	5.9	12.3	20.0
Trimester 2 (14-26w)	17.4	6.2	12.0	22.1
Trimester 3 (27w-delivery)	18.2	6.3	12.8	22.9
Whole pregnancy	17.4	3.6	15.4	19.3

### Predictors and correlates of placental global DNA methylation

Mean (± SD) global DNA methylation levels of placental tissue was 4.6% (± 0.4). First, we identified potential predictors of global DNA methylation levels in placental tissue and/or factors that may confound the association between methylation levels and PM_2.5_ exposure (Table 3). Placental global DNA methylation was lower in girls as compared with boys (β = -0.108 ± 0.049, *p =* 0.03) and was lower in past-smokers [*n* = 39] (β = -0.133 ± 0.067, *p =* 0.05) and smokers [*n* = 39] (β = -0.102 ± 0.068, *p =* 0.13) as compared with never-smokers [*n* = 162] (Reference). Placental methylation levels correlated positively with intake of acetaminophen during pregnancy (β = 0.129 ± 0.049, *p =* 0.009). Placental methylation levels correlated with season at conception; the levels were highest in spring (β = 0.351 ± 0.070, *p* < 0.0001) and lowest in fall (Reference). In addition, placental methylation levels correlated with apparent temperature averaged over the first trimester (β = 0.019 ± 0.004, *p* < 0.0001), second trimester (β = 0.006 ± 0.004, *p* = 0.15) and third trimester (β = -0.021 ± 0.004, *p* < 0.0001).

**Table 3:** **Predictors of placental global methylation in mother-newborn pairs (*n*** **= 240)**

**Variables**	**β**	**SE**	***p*-value**
Maternal characteristics			
Age, y	0.0005	0.005	0.93
Pre-gestational BMI, kg/m^2^	0.002	0.005	0.68
Net weight gain, kg	0.002	0.004	0.54
Maternal education			
Low	Ref	-	
Middle	-0.071	0.081	0.38
High	-0.076	0.077	0.33
Smoking			
Never-smoker	Ref	-	
Past-smoker	-0.133	0.067	0.05
Smoker	-0.102	0.068	0.13
Acetaminophen			
No	Ref	-	
Yes	0.129	0.049	0.009
Alcohol^a^			
No	Ref	-	
Yes	-0.036	0.065	0.58
Parity			
1	Ref	-	
2	0.054	0.053	0.31
≥ 3	0.074	0.085	0.38
Newborn characteristics			
Newborn’s gender			
Male	Ref	-	
Female	-0.108	0.049	0.03
Ethnicity^b^			
European	Ref	-	
Non-European	-0.020	0.074	0.78
Gestational age, w	0.028	0.019	0.15
Birth weight, g	0.0001	0.00006	0.06
Birth length, cm	0.026	0.013	0.05
Time related characteristics			
Season^c^			
Fall	Ref	-	
Winter	0.096	0.067	0.15
Spring	0.351	0.070	< 0.0001
Summer	0.097	0.065	0.14
Apparent temperature, °C			
Trimester 1	0.019	0.004	< 0.0001
Trimester 2	0.006	0.004	0.15
Trimester 3	-0.021	0.004	< 0.0001

β-estimate is an absolute change in percentage of global DNA methylation.

^a^Data available for 235 and ^b^238 subjects.

^c^Season determined for date of conception.

### Placental DNA methylation at birth in association with PM_2.5_ exposure

**Figure 1:**
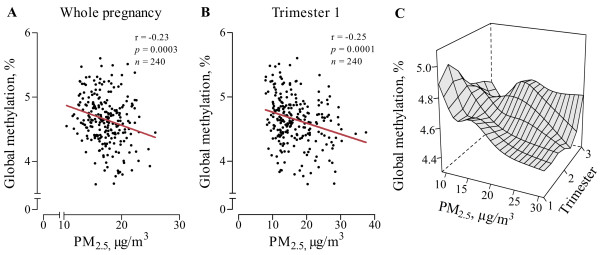
**Bivariate analysis of placental global DNA methylation in association with prenatal exposure to fine particulate air pollution (PM**_**2.5**_**).** PM_2.5_ exposures (μg/m^3^) are presented with spearman correlation coefficients for the whole pregnancy (**A**) and first trimester (**B**). In panel **C**, the association between average PM_2.5_ concentrations and placental global DNA methylation is given. The effect of the average concentrations over 91 days periods was estimated using restricted cubic splines with 5 knots located at the 5^th^, 25^th^, 50^th^, 75^th^ and 95^th^ percentiles.

**Figure 2:**
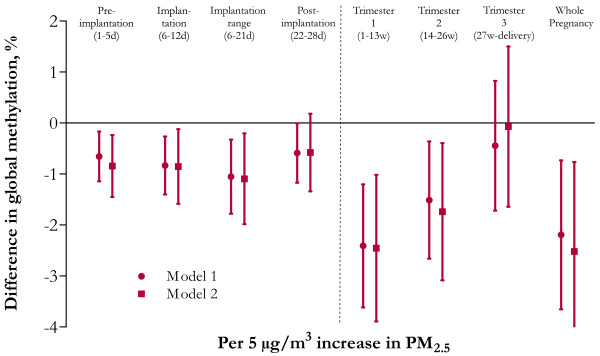
**Relative difference in global DNA methylation of placental tissue in association with *in utero *exposure to particulate air pollution (PM**_**2.5**_**) during various time windows (*n *= 240).** The effect size is a relative difference (95% CI) in mean placental global DNA methylation for each 5 μg/m^3^ increase of PM_2.5_ exposure (μg/m^3^). Model 1 (●) is adjusted for newborn’s gender, maternal age, gestational age, parity, maternal education, smoking status, prenatal acetaminophen use, season at conception and trimester-specific apparent temperature. Model 2 (■) is additionally adjusted for the corresponding NO_2_ and maximum 8-hour average O_3_ exposure.

Although maternal age, maternal education, gestational age and parity were not significantly associated with global DNA methylation, we forced these variables into the multiple regression models, together with newborn’s gender, smoking status, prenatal acetaminophen use, season at conception and trimester-specific apparent temperature. Both before (Figure 1A-C) and after adjustment (Figure 2) for the aforementioned variables, placental global DNA methylation was inversely associated with PM_2.5_ exposures during the whole pregnancy, which was mainly driven by the exposures during the first trimester. Because the inter-quartile range of the PM_2.5_ exposure of the different exposure windows differed only slightly, the reported effect estimates over the different periods were not explained by differences in the window specific distribution of PM_2.5_. Overall, placental methylation relatively decreased by 2.19% (95% confidence interval [CI]: -3.65, -0.73%, *p =* 0.004) for each 5 μg/m^3^ increase in exposure to PM_2.5_. Looking into different exposure windows during pregnancy showed significantly and inverse associations between global DNA methylation at birth with exposures during first trimester (-2.41% per 5 μg/m^3^ increase, 95% CI: -3.62, -1.20%, *p* = 0.0001) and second trimester (-1.51% per 5 μg/m^3^ increase, 95% CI: -2.66, -0.36%, *p* = 0.01), while no significant association was observed with PM_2.5_ exposure of the third trimester (-0.45% per 5 μg/m^3^ increase, 95% CI: -1.72, 0.82%, *p* = 0.49). Further, we ran an additional model in which time-specific exposure windows for PM_2.5_ were combined with time-specific maximum 8-hour average O_3_ and NO_2_ (Figure 2). This did not alter our previous reported findings on PM_2.5_ (effect-size for global methylation relatively decreased by 2.52%, 95% CI: -4.27, -0.76, *p* = 0.005 for each 5 μg/m^3^ increase in PM_2.5_ of trimester 1). Next, we built a multi-lag model in which all the three trimester exposures were fitted as independent variables in the same regression model (Table 4). Only exposure to PM_2.5_ during trimester 1 remained significantly associated with a relative decrease of 2.13% per 5 μg/m^3^ increase (95% CI: -3.71, -0.54%, *p* = 0.009) in global DNA methylation of placental tissue.

**Table 4:** **Relative difference in global DNA methylation in placental tissue in association with *in utero *exposure to particulate air pollution (PM**_**2.5**_**) (*n*** **= 240)**

**Multi-lag model, PM**_**2.5**_^**a, b**^	**Relative difference**	**95% CI**			***p*-value**
Trimester 1 (1-13w)	-2.13%	-3.71	to	-0.54%	0.009
Trimester 2 (14-26w)	-0.43%	-1.84	to	0.98%	0.55
Trimester 3 (27w-delivery)	0.74%	-0.85	to	2.33%	0.36

^a^All the three trimester exposures were fitted as independent variables in the same regression model. The effect size is a relative difference (95% CI) in mean placental global DNA methylation for each 5 μg/m^3^ increase of PM_2.5_ exposure (μg/m^3^).

^b^Adjusted for newborn’s gender, maternal age, gestational age, parity, maternal education, smoking status, prenatal acetaminophen use, season at conception and trimester-specific apparent temperature.

Within trimester 1, we analyzed shorter time windows specifically drawn to target the critical stages of DNA methylation. We observed a decrease of placental global DNA methylation with PM_2.5_ exposure during all the implantation stages (Figure 2) but exposure during the implantation range (6-21d) was strongest associated with placental global DNA methylation at birth (-1.08% per 5 μg/m^3^ increase, 95% CI: -1.80, -0.36%, *p* = 0.004). The association between placental global DNA methylation and PM_2.5_ exposure from the post-implantation window onwards weakens compared with the implantation exposure windows (-0.59% per 5 μg/m^3^ increase, 95% CI: -1.17, -0.005%, *p* = 0.05) and appeared not significant after additional adjustment for the corresponding NO_2_ and maximum 8-hour average O_3_ exposure period (-0.58% per 5 μg/m^3^ increase, 95% CI: -1.34, 0.18%, *p* = 0.14). Studying all different weeks of trimester 1 (week 1–13), we found in addition to the implantation period (Figure 2), a significant inverse association between PM_2.5_ residential exposure estimates for only week 7 adjusted for NO_2_ and maximum 8-hour average O_3_ and placental global DNA methylation (-1.03% per 5 μg/m^3^ increase CI: -1.68, -0.38%, *p* = 0.002).

### Sensitivity analysis

Variation in method of delivery can affect release of stress hormones that may influence methylation [21]. Restricting our analysis to only vaginal partus (excluding 9 caesarean deliveries) did not alter the observed effects for any exposure window. Models in which we replaced the classification of smoking (never-smokers/past-smokers/smokers) by either never-smoker/smoker, pack years or amount of cigarettes during pregnancy showed comparative results. Since the weeks after conception might be particularly critical for DNA methylation, we also evaluated the effect of tobacco smoke on methylation levels of mothers who stopped smoking upon learning of being pregnant but no difference in methylation level was seen (*n* = 11, *p* = 0.28). Finally, we did not observe effect-modification by newborn’s gender (*p* > 0.22) and birth weight (*p* > 0.37) on the association between global DNA methylation and PM_2.5_ during the different exposure windows. We also did not observe effect-modification by trimester-specific apparent temperature on the placental DNA methylation and PM_2.5_ exposure during trimester 1 (interaction term *p* ≥ 0.19).

## Discussion

The placenta plays a pivotal role in nutrient transfer, growth, and organ development of the embryo. Epigenetic modification may provide a plausible link between particulate air pollution and alteration in gene expression that might lead to disease phenotypes related to fetal programming. The key finding of our study is that exposure to particulate air pollution from fertilization up to and including embryo implantation was associated with lower global DNA methylation levels in placental tissue at birth. This observation persisted after adjustment for newborn’s gender, maternal age, gestational age, parity, smoking, maternal education, prenatal exposure to acetaminophen, season at conception, trimester-specific apparent temperature or any other covariate studied.

DNA methylation patterns are established in two developmental periods (germ cells and early embryos) and are likely needed to generate cells with a broad developmental potential and correct initiation of embryonic gene expression [23]. In this regard, epigenetic reprogramming of imprinted genes in germ cells and early embryos appear to be particularly important for the regulation of embryonic growth and placental development [24]. It has been hypothesized that regulation of imprinted gene expression is less stable in the placenta than in the fetus itself which may aid the placenta in adapting to changing physiological conditions [25,26]. This leads to speculation that perturbations in DNA methylation patterns or sporadic loss-of-imprinting in the early stages of development lie at the basis of altered gene expression and contribute to abnormal placental or fetal development [25]. Indeed, research suggests that transplacental exposure to environmental toxicants during critical developmental periods lead to disease pathogenesis in later life [12,14,27,28]. Both in animal and human cells, there is direct evidence for the role of hypomethylation for inducing genomic and chromosomal instability [29-31].

The sensitivity of the epigenetic system to environmental factors occurs primarily during the period of developmental plasticity because this is the time when epigenetic marks undergo critical modifications [32]. After fertilization and prior to implantation, DNA methylation patterns are largely erased but are reestablished by *de novo* DNA methyltransferases (DNMTs) in the blastocyst stage [33]. The placenta develops from the outer layer of the blastocyst upon implantation into the maternal endometrium [34]. Our results show that exposure to particulate air pollution during the implantation window is associated with the methylation profile of placental tissue. The finding of lower methylation levels from the beginning of placental formation is of critical interest in development considering that disturbance of maintenance DNA methylation in placental tissue is associated with abnormal embryonic development in the mouse model [35] and genetic inactivation of DNMTs is lethal to developing mouse embryos [36]. Experimental evidence showed that oxidative DNA damage could interfere with the capability of methyltransferases to interact with DNA resulting in lower methylation of cytosine residues at CpG sites [37]. Since trophoblast differentiation is most important early in pregnancy when the placenta is initially being constructed [38] and maternal air pollution exposure may influence markers of placental growth and function [39], it could well be that altered global DNA methylation during early pregnancy influences placental development. Maternal tobacco smoke, a personalized form of air pollution, has shown to alter placental methylation levels [22,40] and underlie changes to placental function that may lead to altered fetal development and programming [22] or pregnancy pathologies such as impaired fetal growth [41] and preterm delivery [42,43]. Our relative estimates of lower global DNA methylation levels for an increase of 5 μg/m^3^ in the first trimester is associated with a decrease of 2.13% (*p* = 0.009) in global DNA methylation, compared with -2.17% (*p* = 0.13) in active smokers and -2.84% (*p* = 0.05) in past smokers. Our observations in smokers are much smaller compared with the estimates in cord blood assessed by ELISA in the study of Guerrero-Preston and colleagues showing a -48.5% (*p* < 0.01) lower global DNA methylation among newborns with smoking mothers compared with their nonsmoking counterparts [44]. However, differences in tissue and techniques make direct comparison of methylation status difficult. The mechanisms of air pollution-induced health effects involve oxidative stress and inflammation [45,46]. The associations we observed in our current study may be part of the systemic consequences of induced inflammatory conditions both in mother lungs as well as in placental tissue. An alternative hypothesis is that inhaled particles may translocate directly from the lung into the blood stream where these fine particles induce oxidative stress in blood cells and potentially in placental tissue [47,48].

Although this is the first study investigating the effect of PM_2.5_ on DNA methylation in early life, several other studies have examined the role of environmental factors on DNA methylation levels in adults. Baccarelli and colleagues showed that blood DNA methylation in the LINE-1 repetitive element was decreased in elderly individuals of the Normative Aging Study with recent exposure to higher levels of traffic particles including PM_2.5_, whereas no association was observed between methylation of the Alu repetitive element and particle levels [18]. Another study within the same elderly cohort found that prolonged exposure to black carbon and sulfate particles is associated with hypomethylation of Alu and LINE-1 in leukocytes respectively [17]. Besides surrogate markers of global DNA methylation, several studies also report associations of gene-specific DNA methylation in leukocytes and exposure to airborne polycyclic aromatic hydrocarbons and PM [19,27]. In contrast to particulate exposure, arsenic was positively associated with DNA methylation in LINE-1 repeated element in both maternal and fetal leukocytes [49].

A first limitation of this study is that placental tissue is composed of a complex population of cells (syncytiotrophoblasts/cytotrophoblasts, mesenchymal cells, Hofbauer cells, fibroblasts). Also maternal blood is a major constituent of placental tissue which makes that this organ shows high variability in overall DNA methylation compared to other tissues [26]. However, within-placenta variability for several genes showed generally less sample-to-sample variation for DNA methylation than gene expression levels and different placental sites and depths show consistent methylation patterns [21,50]. In our study, the coefficient of variation of global methylation between sample spots from different quadrants of the placenta was 4.5% with an intraclass correlation coefficient (ICC) of 0.25. To minimize the impact of regional differences in methylation patterns within a mother’s placenta, we standardized our method and chose one spot. Most of the methylation variation is not due to sample location but rather cell composition differences between samples. Heterogeneity in cell types in placental tissue may also contribute to inter-individual variation [34]. Global methylation status measured by quantifying 5-mdC and dC using ultra-pressure liquid chromatography in combination with tandem mass spectrometry, gives a good estimate of global methylation because it averages total methylation of all cell types. Although most genes present in the two main cell types of the placenta (cytotrophoblasts and fibroblasts) exhibit similar promoter methylation patterns, some specific genes show differential promoter methylation [51]. Methylation status of placental villi reflects mainly the profile of the cytotropoblast cells. We did not observe any obvious differences in the histology or cell type composition between the fetal samples taken at four standardized sites across the middle region of the placenta (approximately 4 cm away from the umbilical cord) nor between placentas. Regardless of the limitations, the placenta can be used as a proxy for methylation changes in the fetus as it is derived from the outer layer of the blastocyst. The organ has a great plasticity to a range of intrauterine conditions/exposures and the question remains if the fetus is affected in a direct manner or indirectly by adaptations in its function. Variables interfering with placental integrity may predispose to placenta-related gestational complications such as preeclampsia, fetal growth restriction and abruption [52]. Fetuses adapt their mitochondrial structure and metabolism when the supply of nutrients is limited. Changes in mitochondrial DNA content, may represent a biological effect along the path linking air pollution to effects on the unborn. Recently we showed that mitochondrial DNA content in placental tissue, but not cord blood, was influenced by PM_10_ exposure during the last trimester of pregnancy. The effects of these molecular changes must be further elucidated [53]. Secondly, although our results were consistent after multiple adjustments, we cannot exclude the possibility of residual confounding by some unknown factor that is associated with both placental methylation levels and ambient air pollution. Ambient exposure does not account for indoor exposure, but we obtained information on environmental tobacco smoke. Season and apparent temperature were taken into account as epigenetic adaptive changes to season have been reported in aquatic species [54]. We found the highest methylation levels in placental tissue for conceptions at spring and the lowest in fall, which corresponds with observations in blood from adults by Baccarelli and colleagues [18]. Thirdly, the resolution of our interpolation model (4 x 4 km) may not represent perfect PM_2.5_ exposure at the individual level, however our exposure model has good validation statistics with an explained variance higher than 80% [55] and also validation regarding to personal exposure by measuring carbon load in lung macrophages [56]. Our study was not designed to evaluate temporal changes of DNA methylation during pregnancy and may be hampered by the fact that assays of term placentas may not reflect *in vivo* methylation patterns occurring earlier at critical points of development. Nevertheless, our associations were robust and strong in the context of environmental epidemiology.

Generally, two approaches can be performed to analyze DNA methylation, either gene-specific or global analysis. In our study, we chose to measure global DNA methylation instead of surrogate markers of global DNA methylation. Gene-specific assays are crucial for integrating information about DNA methylation patterns with gene expression at promoter level but do not provide a global picture of DNA methylation changes within the genome [57]. Genome-wide methylation assays and gene expression analysis are needed to complement our findings of lower global methylation levels. For example, investigating DNMTs should give more insight into possible mechanisms that control epigenetic programming and thus placental development. Additional studies should also elucidate gene-specific methylation patterns since there is evidence that altered DNA methylation at the human *H19*/*IGF2* imprinting control region [25], genes such as *TIMP3*[58] and disruption of imprinted genes in mouse models may be associated with abnormal placental outcomes and fetal development [59]. Our findings give mechanistic plausibility to the hypothesis that air pollution is linked to fetal programming. Indeed, there is an increasing awareness that the placenta responds to and modulates perturbations in the maternal environment, thereby playing a key role in transmitting the programming stimuli to the fetus [60].

The current study was performed in an European hotspot regarding particulate air pollution [61] with 33 days in 2011 exceeding the European legislation of 50 μg/m^3^. Thanks to legislation, levels of urban air pollution have generally decreased over the course of the latter half of the 20^th^ century in the United States and Western Europe. However, no such trend has taken place in many cities and megacities of developing countries.

## Conclusions

We observed a lower degree of placental global DNA methylation in association with exposure to particulate air pollution during early pregnancy. More specifically, exposure from fertilization up to and including implantation, a critical period for methylation reprogramming, was a highly sensitive window for PM_2.5_ exposure on placental DNA methylation at birth. There is need to further investigate how environmental conditions such as particulate air pollution affect gene-specific DNA methylation and gene expression patterns during fetal development.

## Methods

### Study population and data collection

The Ethics Committee of Hasselt University and South-East-Limburg Hospital approved the protocol of the ENVIR*ON*AGE birth cohort study [53]. Between Friday 1200 hours and Monday 0700 hours from 5 February 2010 until 21 January 2012, we recruited 258 mother-infant pairs (only singletons) in the South-East-Limburg Hospital in Belgium. The catchment area of the hospital includes the province Limburg in Belgium and combines both urban and suburban to rural areas with population densities between the municipalities ranging from 82 to 743 inhabitants/km^2^.

The placenta could not be collected from six newborns, DNA yield was insufficient for ten placentas and two newborns had missing data for PM_2.5_ exposure. We ended with a final sample size of 240 newborns. The only inclusion criterion was that mothers had to be able to fill out questionnaires in Dutch. Enrollment was spread equally over all seasons of the year. The overall participation rate of eligible mothers was 56%. Participating mothers provided written informed consent when they arrived at the hospital for delivery, and completed study questionnaires in the postnatal ward after delivery to provide detailed information on age, pre-gestational body mass index (BMI), maternal education, occupation, smoking status, alcohol consumption, place of residence, use of medication, parity and newborn’s ethnicity. Maternal education was coded as low (no diploma or primary school), middle (high school) or high (college or university degree). Past-smokers were defined as those who had quit before pregnancy and smokers as having smoked before and during pregnancy.

Samples of placental tissue were collected immediately after delivery, along with perinatal parameters such as newborn’s gender, birth date, birth weight and length, gestational age (range, 35–42 weeks), Apgar score, and ultrasonographic data. All neonates were assessed for congenital anomalies immediately after birth and were considered healthy with an Apgar score after 5 min ranging between 7 and 10. No neonate was delivered in the Neonatal Intensive Care Unit. The ENVIR*ON*AGE birth cohort generally consists of mothers with normal pregnancies without complications and with healthy neonates.

### Sample collection

Placentas were obtained from 252 mothers and deep-frozen within 10 minutes after delivery. Afterwards, we thawed placentas to take tissue samples (each biopsy was approximately 1 to 2 cm^3^) for DNA extraction following a standardized protocol as described by Adibi et al. [62]. Briefly, villous tissue, protected by the chorio-amniotic membrane, was biopsied from the fetal side of the placenta and preserved at -80°C. We assessed within-placenta variability in a random subset of seven placentas by comparing biopsies taken at four standardized sites across the middle region of the placenta, approximately 4 cm away from the umbilical cord. The first biopsy was taken to the right of the main artery and the three other biopsies in the remaining quadrants of the placenta. Methylation levels within each placenta varied by a mean of 4.5% (CV) across the quadrants (ICC = 0.25). To minimize the impact of within-placental variability, biopsies were all taken 1–1.5 cm below the chorio-amniotic membrane at a fixed location by using a device to orientate the fetal side of the placenta in relation to the umbilical cord. Care was taken by visual examination and dissection to avoid the chorio-amniotic membrane contamination.

### Exposure estimates

We interpolated the regional background levels of PM_2.5_ for each mother’s residential address using a spatial temporal interpolation method (Kriging) that uses land cover data obtained from satellite images (Corine land cover data set) in combination with monitoring stations (*n* = 34) [55,56,63]. This model provides interpolated PM_2.5_ values from the Belgian telemetric air quality networks in 4 × 4 km grids. Based on 34 different locations, validation statistics of the interpolation tool gave a temporal explained variance (R^2^) for hourly averages PM_2.5_ > 0.80 and a spatial R^2^ for annual mean PM_2.5_ > 0.80 [55]. To explore potentially critical exposures during pregnancy, individual mean PM_2.5_ concentrations (micrograms per cubic meter) were calculated for various periods, for which the date of conception was estimated based on ultrasound data: each of the three trimesters of pregnancy, with trimesters being defined as: 1–13 weeks (trimester 1), 14–26 weeks (trimester 2) and 27 weeks to delivery (trimester 3); and early pregnancy stages, with windows being defined as: 1–5 days (pre-implantation), 6–12 days (implantation), 6–21 days (implantation range) and 22–28 days (post-implantation week 4). Also, the whole pregnancy exposure was calculated as the mean of all pregnancy days. We have complete residential information during and before pregnancy. For those that moved during pregnancy (*n* = 14; 5.8%), we calculated exposure windows accounting for the address changes during the period. Previously, our long-term exposure estimates have been validated by the association between modeled air pollution and carbon load in lung macrophages [56]. Additionally, NO_2_ and maximum 8-hour average O_3_ exposures were interpolated using the same methods as PM_2.5_ exposure.

The Royal Meteorological Institute (Brussels, Belgium) provided mean daily temperatures and relative humidity for the study region which were averaged using the same exposure windows as for PM_2.5_. Apparent temperature was calculated by using the following formula [64,65]: –2.653 + (0.994 × Ta) + (0.0153 × Td^2^), where Ta is air temperature and Td is dew-point temperature (in degrees Celsius).

### Global DNA methylation analysis

Genomic DNA was isolated from placental tissue using the MagMAX™ DNA Multi-Sample kit (Applied Biosystems, Foster City, CA, USA). Mean DNA yield was 80.0 ng/μl with purity values of 1.9 for A260/280 ratio and 2.0 for A260/230 ratio.

We determined global DNA methylation as previously published [66,67]. Briefly, isolated genomic DNA samples were hydrolyzed to individual deoxyribonucleosides in a simplified one-step procedure [68]. A digest mix was prepared by adding 300 mU Phosphodiesterase I (Sigma Aldrich, P3134-100MG), 200 U alkaline phosphatase (Sigma Aldrich, P7923-2KU) and 250 U Benzonase^®^ Nuclease (Sigma Aldrich, E1014-5KU) to 5 ml Tris–HCl buffer (pH 7.9, 20 mM) containing 100 mM NaCl and 20 mM MgCl_2_. Extracted DNA (1 μg diluted in 50 μl HPLC-grade water) was hydrolyzed by adding 50 μl digest mix and incubating at 37°C for 24 h. After hydrolysis, water (HPLC-grade) was added to the samples up to a total volume of 1 ml. Reference standards for 5’-methyl-deoxycytidine (5-mdC) and deoxycytidine (dC) were purchased from Jena Bioscience (N-1044) and Sigma (D3897-1G) respectively. Stock solutions of 5-mdC and dC were prepared by dissolving the purchased solid reference standards in pure water (HPLC-grade). Using these stock solutions, a series of calibration solutions was prepared for 5-mdC and dC in a range of 0.1-10 ng/mL and 1–100 ng/mL respectively. The same calibration standards were used in all of the experiments. Global DNA methylation was obtained by quantifying 5-mdC and dC using ultra-pressure liquid chromatography (UPLC), in combination with tandem mass spectrometry (MS-MS). LC/MS-MS analysis of the samples was conducted on a Waters^®^ Acquity UPLC™, coupled to a Waters^®^ Micromass Quattro Premier™ Mass Spectrometer, using electro spray ionization (ESI). Injections were performed on a Waters^®^ UPLC column (BEH C_18_, 50 mm x 2.1 mm, 1.7 μm) which was held at a temperature of 40°C during analysis.

The global DNA methylation is expressed as a percentage of 5-mdC versus the sum of 5-mdC and dC [5-mdC/(5-mdC + dC)] %. We measured samples in duplicate to account for technical variation which resulted in a R^2^ of 0.8 (ICC = 0.90). The average methylation value of both measurements was used in the statistical analysis.

### Statistical analysis

We used SAS software (version 9.2; SAS Institute Inc., Cary, NC, USA) for database management and statistical analysis. Categorical data are presented as frequencies (%) and numbers, continuous data as mean and standard deviation. Spearman correlation coefficients and linear regression were used to assess the association of global DNA methylation from placental tissue with PM_2.5_. The unadjusted association between the average concentrations over 91 days periods and global methylation was estimated using restricted cubic splines [69] with 5 knots located at the 5^th^, 25^th^, 50^th^, 75^th^ and 95^th^ percentiles. We performed multiple linear regression to determine the independent effect size of PM_2.5_ exposure during pregnancy on global methylation. Covariates considered for entry in the model (*p* ≤ 0.10) using single regression procedures were newborn’s gender, maternal age (years), pre-gestational BMI (kg/m^2^), net weight gain (kg), maternal education (low/middle/high), newborn’s ethnicity (European/non-European), smoking status (never-smoker/past-smoker/smoker), prenatal alcohol use (yes/no), prenatal acetaminophen (yes/no), delivery method (vaginal/caesarean), gestational age (weeks), parity (1/2/≥ 3), season at conception and trimester-specific apparent temperature. Newborn’s gender, maternal age, maternal education, smoking status, gestational age, parity, season at conception and trimester-specific apparent temperature were forced into the model regardless of the *p*-value. In final models we introduced time-specific exposure to NO_2_ and maximum 8-hour average O_3_. Finally, we explored potential effect-modification between trimester-specific PM_2.5_ exposure and birth weight (continuous), newborn’s gender and trimester-specific apparent temperature. Estimates are given as a relative difference from the mean methylation in the whole newborn population. We plotted the studied covariates and global methylation patterns to ensure that there was no threshold phenomenon and that linear regression techniques were appropriate. Q-Q plots of the residuals were used to test the assumptions of all linear models.

## Abbreviations

5-mdC: 5’-methyl-deoxycytidine; dC: deoxycytidine; DNMTs: DNA methyltransferases; ICC: Intraclass correlation coefficient; NO2: Nitrogen dioxide; O3: Ozone; PM2.5: Particulate matter < 2.5 μm.

## Competing interests

The authors declare they have no competing financial interests.

## Authors’ contributions

TSN, WG, BGJ and LG designed the study; BGJ, NP, MP, WG did data collection; KP, LG and BGJ performed analytical measurements; FF and BGJ designed the exposure matrixes; BGJ, MK, and TSN analyzed the data; BGJ and TSN wrote the first draft of paper. All authors critical revised and approved the final version of the manuscript.

## Supplementary Material

Additional file 1: Table S1 Exposure characteristics of NO_2_ and maximum 8-hour average O_3_ (*n* = 240). Click here for file
