# Supplementary material for: Placental DNA hypomethylation in association with particulate air pollution in early life
Source: Part Fibre Toxicol. 2013 Jun 7;10:22. doi: 10.1186/1743-8977-10-22 (PMC3686623; doi:10.1186/1743-8977-10-22)
Supplement: Additional file 1: Table S1 — Exposure characteristics of NO2 and maximum 8-hour average O3 (n = 240). [file 1743-8977-10-22-S1.docx]

**Additional file**

**Placental DNA hypomethylation in association with particulate air pollution in early life**

Bram G. Janssen^1^, Lode Godderis^2,3^, Nicky Pieters^1^, Katrien Poels^2^, Michał Kiciński ^1^, Ann Cuypers^1^, Frans Fierens^4^, Joris Penders^5,6^, Michelle Plusquin^1^, Wilfried Gyselaers^6,7^, Tim S. Nawrot^1,2^

^1^ Centre for Environmental Sciences, Hasselt University, Diepenbeek, Belgium;

^2^ Department of Public Health, Occupational, Environmental & Insurance Medicine, Leuven University (KU Leuven), Leuven, Belgium;
^3^ Idewe, External Service for Prevention and Protection at Work, Heverlee, Belgium;

^4^ Belgian Interregional Environment Agency, Brussels, Belgium;

^5^ Laboratory of Clinical Biology, East-Limburg Hospital, Genk, Belgium;

^6^ Biomedical Research Institute, Hasselt University, Diepenbeek, Belgium;

^7^ Department of Obstetrics, East-Limburg Hospital, Genk, Belgium.

Supplemental Materials, Table 1. Exposure characteristics (*n* = 240).

| Pollution indicator | Mean^a^ | SD | 25^th^ percentile | 75^th^ percentile |
| --- | --- | --- | --- | --- |
| NO_2_, µg/m³ |  |  |  |  |
| Trimester 1 | 21.5 | 6.1 | 16.9 | 24.9 |
| Trimester 2 | 22.1 | 6.6 | 16.8 | 26.5 |
| Trimester 3 | 22.2 | 6.8 | 17.2 | 26.8 |
| Whole pregnancy | 21.9 | 4.9 | 18.8 | 24.8 |
| O_3_, µg/m³a |  |  |  |  |
| Trimester 1 | 63.3 | 19.8 | 44.0 | 80.9 |
| Trimester 2 | 60.1 | 20.8 | 41.0 | 79.2 |
| Trimester 3 | 61.2 | 20.6 | 41.8 | 79.3 |
| Whole pregnancy | 61.5 | 8.0 | 54.9 | 67.5 |
| ^a^Maximum 8-hour average ozone concentration. | | | | |
